# Determination of intracellular antibody production, cell density, and viability of recombinant CHO-DG44 cells using the MACSQuant® Analyzer

**DOI:** 10.1186/1753-6561-5-S8-P97

**Published:** 2011-11-22

**Authors:** Jörn Pluschke, Sandra Klausing, Annika Haseloff, Bernd Schröder, Tanno Hübel

**Affiliations:** 1 Miltenyi Biotec GmbH, Teterow, Germany; 2 TeutoCell AG, Bielefeld, Germany

## Introduction

Expression of recombinant antibodies in CHO cells is a state-of-the-art procedure in research and industry. Generation of cell lines producing high amounts of antibodies is one of the major tasks to increase process efficiency. The establishment of clones is often achieved by methotrexate (MTX)–mediated gene amplification in CHO-DG44 cells. Evaluation of MTX-mediated amplification is usually facilitated by intracellular staining of CHO cells.

We used the MACSQuant® Analyzer to determine the fraction of antibody-producing cells following staining of intracellular IgG with fluorochrome-conjugated antibodies. Additionally, the instrument was utilized for the rapid and reproducible determination of cell density and viability.

## Materials and methods

### Cell density and viability measurements

Cell density and viability were determined by using the MACSQuant Analyzer. The data were compared to those obtained with other commercially available cell-counting devices (Cedex, Roche Diagnostics; Vi-Cell, Beckman Coulter). Dead cell exclusion for flow cytometric analysis with the MACSQuant Analyzer was achieved by propidium iodide (PI) staining. In contrast, the automated cell viability analyzers used video imaging and the trypan blue dye exclusion method for dead cell discrimination.

### Gene amplification in culture

For amplification of the target gene, a transfectant pool of CHO DG44 cells was grown in a perfused 1-L bioreactor with increasing MTX concentrations. Up to 150% of the culture volume was exchanged daily. MTX concentration was doubled every 7 to 14 days depending on cell viability.

### Intracellular IgG staining of CHO cells

Permeabilization of the cell membrane with detergents allowed intracellular staining of the IgG-producing CHO cell pools. IgG^+^ cells were stained with fluorochrome-conjugated antibodies that bind to Fc and kappa chains of IgG within fixed CHO cells.

## Results

### Comparative measurement of cell density and viability using the MACSQuant Analyzer and automated cell viability analyzers

The MACSQuant Analyzer was used to monitor a bioreactor process in comparison to two different automated cell viability analyzers (Cedex, Vi-Cell). We used cells from a fed-batch bioreactor process, cultivated in a benchtop system. Samples were diluted in the same manner for all monitoring systems.

The results for cell density and viability showed good reproducibility in all three systems (see table 1).

**Table 1:** Viable cell density (VCD) and cell viabilities of CHO cells cultured in a fed-batch bioreactor process

	Process time (d)	VCD mean (cells/mL)	STDV	CV (%)	Viability mean (%)	STDV	CV (%)
**Cedex**	0.8	7.33E+05	1.35E+04	1.8	96.39	0.92	1.0
	3.8	4.18E+06	5.94E+04	1.4	97.01	0.33	0.3
							
**MACSQuant Analyzer**	0.8	6.47E+05	9.42E+03	1.5	95.30	0.03	0.0
	3.8	4.41E+06	2.87E+04	0.7	96.06	0.06	0.1
							
**Vi-Cell**	0.8	7.11E+05	1.06E+05	14.9	96.31	0.73	0.8
	3.8	5.03E+06	9.92E+04	2.0	96.98	0.06	0.1

The three methods yielded results with similar general trends, although measurements with the MACSQuant Analyzer showed lower total (and viable) cell densities compared to the Cedex and Vi-Cell instruments. This was likely due to flow cytometric gating of a particular cell population and the use of a threshold to exclude debris. The Cedex and Vi-Cell instruments, however, might still determine small debris particles as cells.

At high cell densities, the standard dilution factor was not suitable for the MACSQuant Analyzer, as it resulted in an elevated events-per-second ratio. Therefore, higher dilutions were applied, which allowed better comparability to the other analyzers (data not shown). Cell viability results obtained with the MACSQuant Analyzer were similar to the results from automated cell analyzers, even though the measurement is based on a different method.

### Gene amplification monitoring by intracellular staining

Intracellular IgG production was used as an indirect parameter to monitor MTX-induced gene amplification. Usually, gene amplification is performed at a clonal level. We followed a different, possibly less time-consuming approach using cell pools.

**Figure 1:**
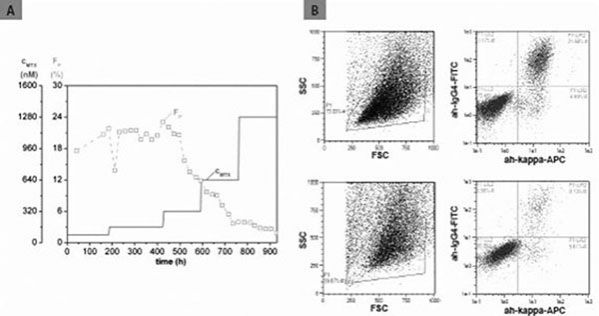
(A) The time course of an unstable MTX amplification is presented. A decrease in the fraction of IgG-producing cells (F_P_) is caused by resistance to increasing MTX concentrations (c_MTX_). (B) Dot plots for the samples at 307 h (160 nM MTX) and 687 h (640 nM) are shown. IgG-producing cells appear in the upper right gates.

Figure 1 shows an example of an unstable cell pool, displaying a decrease in the amount of IgG-producing cells, beginning at the third amplification step (320 nM MTX). The fraction of IgG-producing cells declined from 20% to about 12%. Later on, this trend continued to a final 2%.

Another gene amplification was more stable with regard to the intracellular antibody content. There was an increase in the fraction of IgG-producing cells at the beginning, followed by a stable phase with fractions of more than 90% IgG-producing cells throughout the whole amplification (data not shown). This gene amplification allowed us to generate a high-producer pool in a perfused bioreactor. In contrast, the amplification shown in figure 1 did not yield a high-producer pool as non-producing cells overgrew the producing cells.

## Discussion

The MACSQuant Analyzer is a powerful tool for the evaluation of gene amplification. Intracellular staining provides an in-depth insight into the stability of the cell pool and allows the assessment of amplification efficiency. Despite high concentrations of MTX, a transfectant pool with 20% IgG-producing cells was overgrown by non-producing cells. This may be due to a saturation effect of MTX [1] or to an MTX resistance, caused, e.g., by altered MTX transport properties [2]. In contrast, another pool with 90% IgG-producing cells yielded successful selection of IgG-producing cells and increased IgG titers. Amplification with MTX in a cell pool with a low starting content of IgG-producing cells is not effective.

The MACSQuant Analyzer allows the determination of cell density and viability with high reproducibility. The mean data were comparable to common cell-counting systems. The results obtained with the MACSQuant Analyzer, however, showed better accuracy as reflected by the lower CV. Moreover, through its FSC, SSC, and seven fluorescence channels, as well as its three lasers it allows sophisticated, multiparametric flow cytometric analysis.
